# Effects of Compound Probiotic Fermented Feed on In Vitro Rumen Fermentation, In Situ Degradation, Rumen Microbiota and Metabolome, and Growth Performance of Beef Cattle

**DOI:** 10.3390/metabo16070457

**Published:** 2026-06-29

**Authors:** Haitao Hu, Yuwa Cao, Mei Tian, Hongrui Li, Zhaokun Liu, Thant Mon Paing, Huilin Ma, Siyu Feng, Ruiting Zhang, Dangdang Wang, Lamei Wang, Yangchun Cao

**Affiliations:** 1 College of Animal Science and Technology, Northwest A&F University, Yangling 712100, China; huhaitao711@nwafu.edu.cn (H.H.); 2023055384@nwafu.edu.cn (Y.C.); tianmei@nwsuaf.edu.cn (M.T.); lihongrui02072024@nwafu.edu.cn (H.L.); thantmonpaing12@gmail.com (T.M.P.); 13227792397@nwafu.edu.cn (H.M.); fsy.512@nwafu.edu.cn (S.F.); wangdang@nwafu.edu.cn (D.W.); 2 Department of Animal Science, Yezin Agricultural University, Nay Pyi Taw 150501, Myanmar

**Keywords:** beef cattle, compound probiotic fermented feed, rumen fermentation, metabolomics, growth performance

## Abstract

**Background/Objectives:** This study evaluated the effects of a compound probiotic fermented feed (CPFF) containing *Lactobacillus plantarum*, *Bacillus subtilis*, yeast, and *Aspergillus niger* on rumen in vitro fermentation, in situ feed degradation, and growth performance in beef cattle. **Methods:** We established a control group (CON) and experimental groups with 2%, 4%, and 8% CPFF supplementation for in vitro fermentation. **Results:** The results indicated that the NH_3_-N concentration in the 4% CPFF group was significantly higher than in the other groups (*p* < 0.001). Similarly, microbial crude protein (MCP) production was significantly greater in the 4% CPFF group compared to the CON group (*p* = 0.016). The molar proportions of acetate, butyrate, isobutyrate, and valerate were significantly higher in the 2% and 4% CPFF groups than in the control group (*p* < 0.001), while propionate levels were significantly lower (*p* < 0.001). After 48 h, gas production was highest in the 4% CPFF group. Based on improvements in gas production, MCP synthesis, and fermentation intensity, the 4% inclusion level was determined to be optimal for further studies. We conducted an in situ degradation trial using 4% CPFF. Results showed that at 12 h, the neutral detergent fiber (NDF) degradation rate in the 4% CPFF group was significantly higher than in the CON group at 4, 8, 12, and 48 h (*p* < 0.05). At 48 h, the acid detergent fiber (ADF) degradation rate in the 4% CPFF group was also significantly higher than in the CON group (*p* < 0.001), and this group exhibited a significant increase in crude protein (CP) degradation (*p* = 0.030). We analyzed rumen fluid samples from both the CON and 4% CPFF groups after in vitro fermentation using 16S rRNA sequencing and untargeted metabolomics. Microbial community analysis revealed significantly increased abundances of functional bacterial groups such as *Rikenellaceae_RC9_gut_*group, *Christensenellaceae_R-7_*group, and *UCG-002* in the 4% CPFF group (*p* < 0.05). Differential metabolites were primarily involved in pathways related to tryptophan metabolism, and tyrosine metabolism signaling. A feeding trial was conducted by adding 4% CPFF to the diet of Angus growing cattle. The results indicated that average daily gain (ADG) (*p* = 0.004) and average daily feed intake (ADFI) (*p* = 0.001) were significantly higher in the CPFF group than in the CON group. **Conclusions:** In conclusion, our results demonstrate that CPFF enhances rumen fermentation activity, optimizes the microbiota and metabolic profiles of rumen fluid, and improves the average daily gain of beef cattle. This research provides a valuable theoretical basis for applying CPFF in beef cattle breeding.

## 1. Introduction

Compound probiotic fermented feed (CPFF) refers to a functional feed produced via the directional fermentation of plant-based feed ingredients or agricultural by-products using two or more beneficial microorganisms with complementary functions [1,2]. The fermentation process can pre-digest feed and eliminate anti-nutritional factors, thereby improving feed bioavailability [3,4]. Fermented feed provides probiotics and abundant bioactive metabolites, which help stabilize the gastrointestinal microecology of animals, thereby optimizing their production performance and health status [5,6].

*Lactobacillus plantarum* is among the most widely used strains in the fermented feed industry. These strains utilize feed nutrients as fermentation substrates, metabolizing them to produce significant amounts of lactic acid. Continuous lactic acid production inhibits the growth of harmful pathogens and spoilage microorganisms in the fermentation system, while improving feed storage stability [7]. Bacillus species have the function of producing large amounts of protease, phytase, and cellulase and have been widely used in various fermented foods [8]. Additionally, *Bacillus subtilis* consume oxygen in the gastrointestinal tract, forming an anaerobic microenvironment that facilitates the colonization and proliferation of beneficial anaerobic bacteria within the gut [9]. Additionally, Yeasts enhance the activity of fibrolytic microbial communities by modulating the anaerobic environment in the rumen, which improves roughage digestibility [10]. Furthermore, filamentous fungi such as *Aspergillus niger* decompose tough cell wall structures of feed ingredients and release intracellular nutrients due to their exceptional ability to secrete various lignocellulolytic enzymes [11,12]. Research on individual strains has led to a shift in the development of fermented products toward multi-strain, synergistic fermentation. In fermented beverages, the co-fermentation of Lactobacillus plantarum and yeast exhibits a more pronounced effect compared to single-strain fermentation [13]. Similarly, in plant-based milk fermentation, the mixed fermentation of two or more microorganisms (such as lactic acid bacteria, bacilli, and yeast) is expected to exert synergistic effects, thereby improving the quality of the final product [14]. Furthermore, in vitro fermentation studies have demonstrated that the combined supplementation of *Lactobacillus* and Yeast optimizes rumen microbial populations and hydrolases while simultaneously reducing methane production [15].

It is essential to conduct multi-level supplementation trials to determine the optimal inclusion level of fermented feed in diets for efficient conversion and practical application in ruminant production. In vitro fermentation and fistulated animal trials are important methods for assessing feed fermentation characteristics in ruminants [16], enhancing the efficiency and sustainability of production. The analysis of feed nutrient degradation efficiency, in vitro gas production kinetics, and volatile fatty acid generation serves to evaluate the effects of feed fermentation, while simultaneously offering valuable theoretical insights for nutrient supply levels in feed formulation [17,18]. Although numerous studies show that fermented feed regulates the rumen microbial community structure in ruminants, the specific coupling mechanism between the microbiome and metabolome remains unclear [19]. The primary novelty lies in the systematic, multi-stage validation approach—integrating in vitro, in situ, and in vivo models—to elucidate the positive impacts of the composite probiotic fermented feed on rumen fermentation characteristics and beef cattle growth performance. This study is of significant theoretical importance for elucidating the mechanisms by which CPFF affects rumen fermentation and feed utilization efficiency in ruminants, while also providing a reference for the development of new CPFF.

This study systematically investigated how a CPFF composed of *Lactobacillus plantarum*, *Bacillus subtilis*, Yeast, and *Aspergillus niger* affects rumen fermentation and beef cattle growth performance. A multi-stage experimental design was employed to evaluate these effects. First, in vitro fermentation trials tested various supplementation levels to determine the optimal CPFF dosage. Next, an in situ nylon bag experiment used a rumen-fistulated model to assess how CPFF influences nutrient degradation kinetics. Finally, a feeding trial on growing cattle validated the actual impact of the optimal dosage on growth performance. Based on the synergistic potential of these four microbes, we hypothesized that the optimal dosage of CPFF would shift rumen microbial fermentation toward a more efficient pattern and accelerate nutrient digestion, thereby leading to improved feed efficiency and daily weight gain in beef cattle.

## 2. Materials and Methods

### 2.1. Preparation of CPFF

The CPFF used in the experiment was obtained from a standard production line at Shaanxi Yangling Fushite Bio-Tech Co., Ltd. (Yangling Demonstration Zone, Xianyang, China). The ingredients included in the formulation of CPFF are shown in Table 1. The inoculation ratios include 5% *Lactobacillus plantarum*, 5% *Bacillus subtilis*, 5% *Aspergillus niger*, and 1% Yeast. The process starts with an initial moisture content of 60%, followed by solid-state fermentation at room temperature for 72 h. The product is then air-dried at low temperatures to below 12% moisture, ground, and sieved through a 40-mesh screen. Quality standards require *Bacillus subtilis* ≥ 1 × 10^7^ CFU/g, Yeast ≥ 1 × 10^8^ CFU/g, crude protein ≥ 15.00%, NDF ≤ 18.50%, ADF ≤ 12.80%, mannan ≥ 1.0%, pH of 3.80–4.20, and no mycotoxin contamination. Although the product is manufactured under standardized processes that are expected to minimize batch-to-batch variability, the absence of variations was not empirically confirmed in this study.

### 2.2. Design of In Vitro Rumen Fermentation Experiment

The in vitro fermentation experiment was conducted using a single-factor gradient design experiment, focusing on the inclusion level of CPFF in the concentrate as the experimental factor. The treatments included a control group (CON) without CPFF supplementation and three CPFF supplementation groups: 2% CPFF, 4% CPFF, and 8% CPFF, based on the dry matter of the concentrate. Each treatment was replicated eight times, and two blank bottles without substrate were included to correct gas production values. The experimental unit for the in vitro fermentation test is the fermentation bottle. For each fermentation bottle, 0.50 g of air-dried substrate was accurately weighed and incubated with 60 mL of artificial buffered saliva. The following basic stock solutions were prepared separately. Solution A (elemental nutrient solution) was composed of 13.2 g of CaCl_2_·2H_2_O, 10.0 g of MnCl_2_·4H_2_O, 1.0 g of CoCl_2_·6H_2_O, and 8.0 g of FeCl_3_·6H_2_O, which were dissolved and diluted to a final volume of 1 L with distilled water. Solution B (buffer solution) contained 4 g of NH_4_HCO_3_ and 35 g of NaHCO_3_, also made up to 1 L with distilled water. Solution C (macroelement nutrient solution) consisted of 5.7 g of anhydrous Na_2_HPO_4_, 6.2 g of KH_2_PO_4_, and 0.6 g of MgSO_4_·7H_2_O, and was adjusted to 1 L with distilled water. Solution D (anaerobic indicator nutrient solution) was prepared by dissolving 1 g of crystal violet in distilled water and diluting to 1 L. Solution E (reducing agent nutrient solution) was prepared by dissolving 6.25 g of Na_2_S·9H_2_O in distilled water and diluting to a final volume of 250 mL. To prepare the working solution, 333.0 mL of solution A, 333.0 mL of solution B, and 166.5 mL of solution C were mixed thoroughly. The mixture was then diluted with water to a volume of 841 mL, after which a continuous stream of CO_2_ was bubbled through until the solution became completely colorless. Subsequently, 0.5 mL of solution D was added, followed by the gradual addition of a small amount of solution E until the system turned colorless. Finally, the mixture was brought to a final volume of 1 L with distilled water to obtain the artificial buffer saliva working solution. The bottles were sealed and incubated anaerobically at 39 °C for 48 h. At the end of incubation, fermentation was terminated by placing the bottles in an ice-water bath for 5 min. The ingredient composition and nutrient levels of the concentrate substrate are presented in Table 2.

### 2.3. Donor Animals for Rumen Fluid Collection and Ethical Statement

Rumen fluid was collected from three healthy Qinchuan beef cattle with similar body weights. The donor cattle were fed a total mixed ration (TMR) formulated to meet maintenance and production requirements; the dietary formulation and nutritional levels are detailed in Table 3. The cows were fed twice daily at 07:00 and 17:00 with ad libitum access to water. Rumen fluid was sampled from fasting cows prior to the morning feeding. All animal experimental procedures were approved by the Animal Care and Use Committee of Northwest A&F University (Approval No. NWAFU-DK2024016).

### 2.4. In Vitro Fermentation Procedure and Gas Production Measurement

Gas production was measured at 3, 6, 9, 12, 24, and 48 h of incubation using a petroleum jelly-lubricated 100 mL medical syringe. At each interval, the cumulative gas volume was recorded. The collected gas was then transferred into collection bags to determine methane proportion via gas chromatography (Fuli Instrument Co., Ltd., Wenling, China). Using high-purity nitrogen or hydrocarbon-free air to dilute the high-purity methane, the high-concentration cylinder reference gas was diluted online into a five-point standard series via a dynamic gas diluter (Qingdao Junray Intelligent Instrument Co., Ltd., Qingdao, China). The concentration range properly covered and appropriately extended beyond the expected range of the samples. Each sample was measured in triplicate. Finally, the total gas production volume was calculated according to the formula as described [20].

GP = B(1 − e^−ct^)

In this formula, GP represents the cumulative gas production (mL) at time t, and B denotes the theoretical maximum gas production (mL). The term e is the mathematical constant (e ≈ 2.71828), while c signifies the fractional rate constant of gas production (h^−1^) over the incubation time t (h).

### 2.5. Chemical Composition of Feed and Determination of Rumen Fermentation Parameters

Feed samples were analyzed for crude protein (CP), ether extract (EE), and ash according to GB/T standards 6432–2018, 6433–2006, and 6438–2007 [21,22,23], respectively. Neutral detergent fiber (NDF) and acid detergent fiber (ADF) contents were determined using the method described by Van Soest et al. [24]. Following fermentation, the fluid was filtered through 40–60 μm pore-size nylon bags, and the pH was measured immediately. For volatile fatty acid (VFA) analysis, samples were stabilized with 25% metaphosphoric acid (*v*/*v*) and stored at −20 °C until analysis via gas chromatography (Agilent 7820A GC, Agilent Technologies, Inc., Santa Clara, CA, USA). Additionally, ammonia nitrogen (NH_3_-N) concentration was determined using the phenol–hypochlorite colorimetric method [25]. Finally, microbial crude protein (MCP) (W041-1-1) and lactic acid concentrations (A019-2-1) were quantified using commercial assay kits (Nanjing Jiancheng Bioengineering Institute, Nanjing, China). The determination of the standard curve and sample optical density (OD) values strictly followed the manufacturer’s instructions. Each sample was measured in triplicate. Following instrument startup and preheating, the microplate reader was calibrated, and the OD values were determined using a standard 450 nm filter.

### 2.6. In Situ Rumen Degradation Trial

In situ rumen degradation of the concentrate was evaluated using the nylon bag technique as described by Oliveira [20]. Three healthy sheep, fitted with permanent rumen cannulas, were adapted to a standard diet for seven days prior to the experiment. The substrates consisted of the control concentrate and a concentrate supplemented with the optimal CPFF dosage. Before use, nylon bags (30 μm pore size; 60 mm × 80 mm) were dried at 65 °C to a constant weight and weighed to an accuracy of 0.0001 g. Approximately 3.00 g of each sample was accurately weighed into the bags. All 72 bags (2 groups × 6 time points × 2 replicates × 3 fistulated sheep) were simultaneously placed into the rumen before morning feeding and secured to the cannula cap. The incubation periods were 2, 4, 8, 12, 24, and 48 h. At each interval, the bags were removed and immediately immersed in ice water to stop fermentation. They were then rinsed with tap water until the effluent was clear and dried at 65 °C to a constant weight. Finally, degradability was calculated for dry matter (DM), crude protein (CP), NDF, ADF, and starch. Dry matter degradability (DMD) was determined using the following formula:

$$\mathrm{DMD}=\frac{\left[m_{1}-\left(m_{2}-m_{0}\right)\right]}{m_{1}}\times 100\%$$

In this formula, m_0_ is the weight of the empty nylon bag, m_1_ is the weight of the bag plus substrate before incubation, and m_2_ is the weight of the bag plus residue after incubation.

The degradation rates and effective degradability of feed nutrients were calculated using the rumen kinetics mathematical model as described [26].

Dp = a + b(1 − e − ct);

ED = a + (b × c)/(k + c)

In this formula, a represents the rapidly degradable fraction (%); b represents the slowly degradable fraction (%); c is the degradation rate constant of fraction b (h^−1^); Dp is the rumen degradation rate of a component after an incubation time t; ED is the effective ruminal degradability (%); k is the rumen outflow rate constant, set at 0.05 h^−1^.

### 2.7. Feeding Trial with Growing Cattle

Thirty healthy Angus cattle (age: 17 ± 1 months; weight: 378 ± 22.29 kg) were randomly assigned to two groups, with 15 cattle in each group. The control group (CON) received a basal Total Mixed Ration (TMR) diet. The experimental group (CPFF) was supplemented with 150 g of CPFF per animal daily, representing a 4% inclusion rate in the concentrate. The study spanned 47 days, beginning with a 14-day adaptation phase followed by a 33-day experimental period. Daily feed intake was recorded, and body weights were measured after a 12-h fast at both the start and end of the trial. These data were used to calculate average daily gain (ADG), average daily feed intake (ADFI), and the feed-to-gain ratio (F/G). Details regarding the basal TMR diet’s ingredients and nutrient levels are provided in Table 4.

### 2.8. 16S rRNA Gene Sequencing

Total genomic DNA was extracted using the E.Z.N.A. Bacterial DNA Kit (Omega Bio-tek, Norcross, GA, USA) following the manufacturer’s protocol. The V3–V4 region of the 16S rRNA gene was then amplified with universal primers 338F (5′-ACT CCT ACG GGA GGC AGC AG-3′) and 806R (5′-GGA CTA CHV GGG TWT CTA AT-3′). Subsequently, sequencing libraries were prepared and processed on the Illumina MiSeq platform (San Diego, CA, USA) by Shanghai Majorbio Bio-Pharm Technology Co., Ltd. (Shanghai, China). The sequencing depth per sample averaged 50,000 clean reads, with a minimum of 40,000 clean reads. Bioinformatics analysis, including quality control, denoising, and taxonomic assignment, was performed using QIIME 2 (version 2022.2) via the DADA2 plugin. The raw data after quality filtering was compared to the Greengene 13 database for further analysis. This pipeline followed the workflow established in previous research [27].

### 2.9. Rumen Untargeted Metabolomics

Prior to metabolite extraction, a stable isotope-labeled internal standard mixture, which included L-2-chlorophenylalanine (at a final concentration of 20 µg/mL), was added to all samples. 100 µL of rumen fluid was mixed with 400 µL of extraction solution in a centrifuge tube and vortexed for 30 s. The mixture was incubated at −20 °C for 30 min, then centrifuged at 13,000× *g* for 15 min at 4 °C. The resulting supernatant was collected and evaporated to dryness under nitrogen. The sample was reconstituted in 100 µL of reconstitution solution (acetonitrile–water = 1:1, *v*/*v*), followed by low-temperature ultrasonic extraction for 5 min (5 °C, 40 kHz). After centrifugation at 13,000× *g* and 4 °C for 10 min, the supernatant was transferred to an autosampler vial with an insert for subsequent analysis. Subsequently, metabolite information was acquired using a Thermo UHPLC-Q Exactive HF-X system (Thermo Fisher Scientific, Waltham, MA, USA) equipped with an ACQUITY HSS T3 column in both positive (ESI^+^) and negative (ESI^−^) electrospray ionization modes. The mass spectrometry scan range was set to *m*/*z* 20–1200, with an acquisition speed of 4 Hz. During the analysis process, pooled quality control (QC) samples composed of equal amounts of all research samples were injected to monitor the stability of the system. The aforementioned isotope-labeled internal standard mixture was utilized to correct for ion suppression effects and batch-to-batch variability. Prior to each analytical batch, system suitability was verified using a standard mixture to assess chromatographic separation and mass spectrometer response. Data normalization was performed using MetaboAnalyst 5.0. This was achieved by dividing the peak area of each feature by that of the internal standard with the closest elution time within the same sample. Following normalization, features with missing values exceeding 50% across all samples were excluded from subsequent analyses.

### 2.10. Statistical Analysis

All experimental data were analyzed using SPSS software (version 26). In the experiment, data analysis between two treatment groups was evaluated using the independent samples *t*-test, while comparisons among more than two groups were analyzed using one-way ANOVA. A two-way repeated measures ANOVA and Tukey’s post hoc test were used for multiple comparisons of gas production and in situ degradation data obtained at different time points. Results are expressed as means ± standard error of the mean (SEM). Statistical significance was declared at *p* < 0.05.

Microbial α-diversity indices and relative abundances were analyzed using the Wilcoxon rank-sum test. Microbial community structure was assessed by principal coordinates analysis (PCoA) based on Bray–Curtis distances. The Wilcoxon rank sum test, as a non-parametric test method suitable for comparing two sets of non-normal distributed microbial group data, was used to determine the significance of the microbial genera in the two groups. Correlations between rumen microorganisms, fermentation parameters, and metabolites were evaluated using Spearman’s correlation analysis.

Metabolomics data analysis utilized partial least squares-discriminant analysis (PLS-DA) via the R package ropls (v1.6.2). Differential metabolites were defined by a variable importance in projection (VIP) > 1 and a false discovery rate (FDR) < 0.05. Finally, metabolic pathways were annotated and enriched using the Kyoto Encyclopedia of Genes and Genomes (KEGG) database (https://www.kegg.jp/kegg/pathway.html, accessed on 21 July 2025). The Spearman correlation analysis was used to evaluate the correlations between the differential rumen microorganisms and fermentation parameters, as well as metabolites.

## 3. Results

### 3.1. The In Vitro Rumen Fermentation Characteristics of CPFF

The effects of varying CPFF concentrations on in vitro rumen fermentation parameters are summarized in Table 5. While CPFF supplementation did not influence pH (*p* > 0.05), it significantly altered several key fermentation indicators. Methane production varied across treatments (*p* < 0.001), with the 2% and 4% CPFF groups exhibiting higher levels than the control and 8% groups. Additionally, the 4% CPFF group showed the highest ammonia nitrogen (NH_3_-N) concentration among all treatments (*p* < 0.001). Microbial crude protein (MCP) production was also significantly elevated in the 4% CPFF group compared to the control (*p* = 0.016).

Regarding volatile fatty acids (VFAs), 4% CPFF supplementation significantly increased the acetate-to-propionate ratio (A/P) compared to the control (*p* < 0.001). Conversely, the 8% CPFF treatment significantly reduced the concentrations of propionate and isovalerate (*p* < 0.05). In the 2% and 4% CPFF groups, the molar proportions of acetate, butyrate, isobutyrate, and valerate were significantly higher than in the control group, whereas the proportion of propionate was significantly lower (*p* < 0.001).

### 3.2. Gas Production Kinetics

Gas production kinetics over 48 h are detailed in Table 6. At all recorded time points (6, 9, 12, 24, and 48 h), cumulative gas production was significantly higher in the 2% and 4% CPFF groups than in the control and 8% CPFF groups (*p* < 0.001). Specifically, the 4% CPFF group reached the maximum gas volume of 141.13 mL/g at 48 h. The theoretical maximum gas production (B) followed this trend, peaking in the 4% CPFF group at 148.92 mL/g (*p* < 0.001). Additionally, the gas production rate constant (c) was significantly higher in the 4% CPFF group (0.088%/h) compared to the CON and 8% CPFF groups (*p* = 0.002). Consequently, due to the overall improvements in gas production, MCP synthesis, and fermentation intensity, the 4% inclusion level was selected as the optimal dosage for subsequent studies.

### 3.3. In Situ Nutrient Degradability

Table 7 presents the in situ degradation kinetics comparing the 4% CPFF supplemented group to the CON group. While the effective degradability (ED) of dry matter (DM) did not differ significantly between groups (*p* = 0.311), the 4% CPFF group showed a significantly higher DM degradation rate at 12 h (*p* = 0.004). As for starch, the ED value of the 4% CPFF group was lower than that of the CON group (*p* = 0.034). Conversely, CPFF supplementation markedly enhanced fiber degradation. NDF degradation rates at 4, 8, 12, and 48 h were significantly higher in the 4% CPFF group (*p* < 0.05), raising the NDF ED from 29.87% to 35.67% (*p* = 0.003). Similarly, the ED value of ADF in the 4% CPFF group was higher (*p* = 0.017). Finally, although 4% CPFF significantly increased the crude protein (CP) degradation rate at 8 h (*p* = 0.008), the overall ED of CP remained statistically similar between the two groups (*p* = 0.643).

### 3.4. Rumen Microorganisms

**Figure 1:**
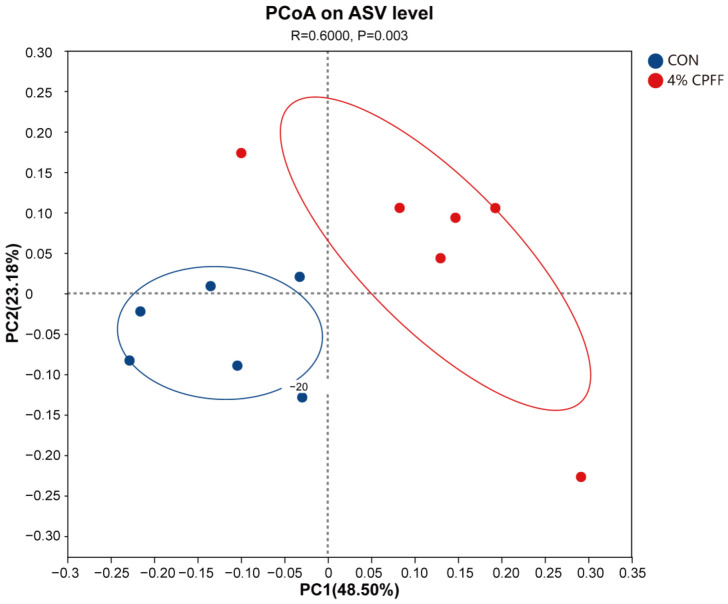
PCoA plot of microbiota.

**Figure 2:**
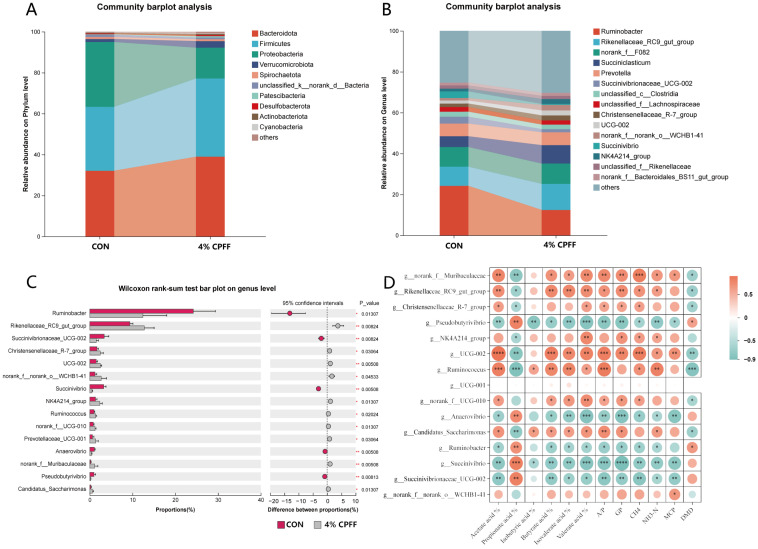
Analysis of rumen microbial community composition in the CON and 4% CPFF groups. (**A**) Relative abundance of rumen bacteria at the phylum level; (**B**) Relative abundance of rumen bacteria at the genus level; (**C**) Wilcoxon rank-sum test bar plot identifying significant genus-level differences between the two groups; (**D**) Spearman correlation heatmap between rumen microorganisms and fermentation parameters. * *p* < 0.05, ** *p* < 0.01, *** *p* < 0.001, **** *p* ≤ 0.0001; blank cells indicate no significant correlation (*p* ≥ 0.05).

Table 8 presents the alpha diversity indices for rumen microorganisms in beef cattle. Compared to the CON group, the 4% CPFF group exhibited significantly higher Shannon, Chao1, Sobs, and ACE indices (*p* < 0.05), alongside a significantly lower Simpson index (*p* = 0.015). These results indicate that CPFF supplementation increased microbial richness and diversity. Furthermore, Principal Coordinate Analysis (PCoA) based on Bray–Curtis distances (Figure 1) showed a distinct separation between the bacterial communities of the two groups, confirming that CPFF reshaped the rumen microbiota structure. At the taxonomic level, Bacteroidota, Firmicutes, and Proteobacteria dominated the microbial communities at the phylum level, while Ruminobacter, Rikenellaceae_RC9, and Prevotella were the predominant genera (Figure 2A,B).

At the genus level, the relative abundances of Ruminobacter (*p* = 0.013), Succinivibrionaceae_*UCG-002* (*p* = 0.008), Succinivibrio (*p* = 0.005), and Anaerovibrio (*p* = 0.005) were significantly higher in the CON group (Figure 2C). In contrast, the 4% CPFF group showed significant increases in *Rikenellaceae_RC9_gut_*group (*p* = 0.008), *Christensenellaceae_R-7_*group (*p* = 0.031), *UCG-002* (*p* = 0.005), Ruminococcus (*p* = 0.020), and Prevotellaceae_UCG-001 (*p* = 0.031). Correlation analysis (Figure 2D) revealed that the genera enriched by 4% CPFF, specifically *Rikenellaceae_RC9_gut_*group and *UCG-002*, were positively correlated with acetate, butyrate, and gas production, but negatively correlated with propionate. Similarly, *Christensenellaceae_R-7_*group showed positive correlations with acetate and gas production, and a negative correlation with propionate. Conversely, genera that decreased in the 4% CPFF group such as Succinivibrionaceae_*UCG-002*, Succinivibrio, and Anaerovibrio were negatively correlated with acetate, butyrate, and gas production.

### 3.5. Rumen Metabolomics

**Figure 3:**
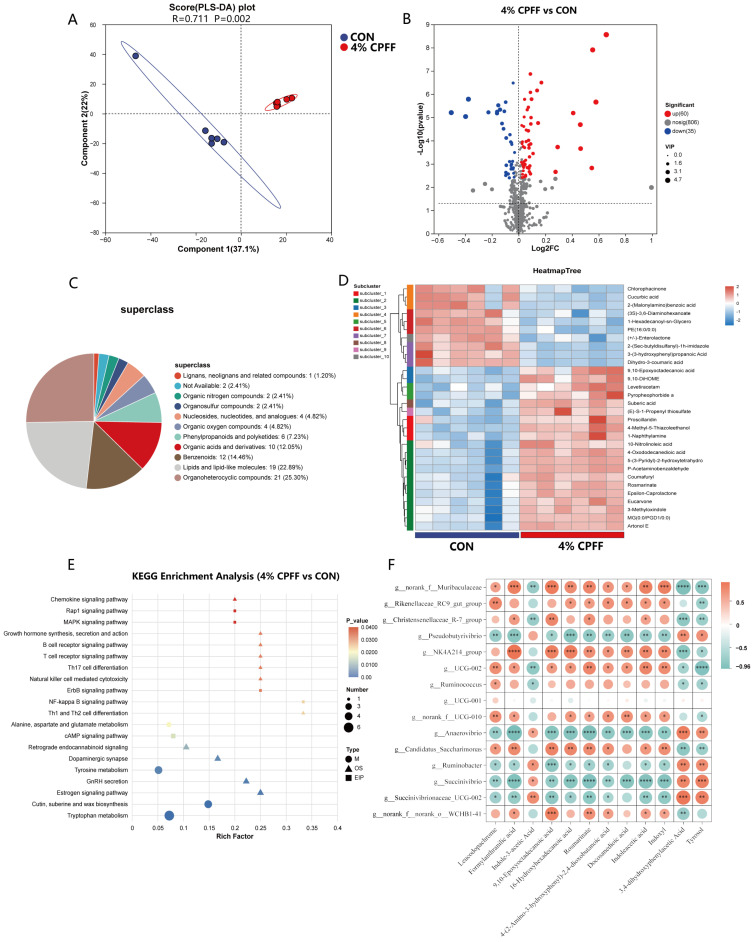
Metabolomic analysis of rumen fluid in the CON and 4% CPFF groups. (**A**) PLS-DA score plot of rumen metabolites; (**B**) Volcano plot showing differential metabolites (4% CPFF vs. CON); (**C**) Classification of metabolites by HMDB compound classes; (**D**) Cluster analysis heatmap of differential metabolites; (**E**) KEGG pathway enrichment analysis; (**F**) Spearman correlation analysis between metabolites and microbial genera. * *p* < 0.05, ** *p* < 0.01, *** *p* < 0.001, **** *p* ≤ 0.0001; blank cells indicate no significant correlation (*p* ≥ 0.05).

The PLS-DA plot demonstrates distinct metabolic profiles between the CON and 4% CPFF groups (Figure 3A). Volcano plot analysis identified 95 differential metabolites, consisting of 60 upregulated and 35 downregulated compounds (Figure 3B). According to the HMDB compound classification, these metabolites were primarily distributed among superclasses such as organic acids and derivatives, benzenoids, lipids and lipid-like molecules, and organoheterocyclic compounds (Figure 3C). Furthermore, these differential metabolites exhibited significant clustering (Figure 3D). KEGG pathway enrichment analysis revealed that the metabolites were mainly involved in the estrogen signaling pathway and various amino acid metabolic pathways, including tryptophan, tyrosine, and alanine, aspartate, and glutamate metabolism (Figure 3E). Correlation analysis (Figure 3F) further showed that leucodopachrome and rosmarinate were significantly positively correlated with *Rikenellaceae_RC9_gut_*group and *UCG-002*, respectively. Additionally, formylanthranilic acid and indoxyl both displayed significant positive correlations with the genus *UCG-002*.

Table 9 lists the key differential metabolites identified within the affected metabolic pathways. In the 4% CPFF group, leucodopachrome and rosmarinate both associated with tyrosine metabolism were significantly upregulated. Similarly, the tryptophan metabolism pathway showed an upregulation of indoleacetic acid, formylanthranilic acid, and indoxyl. Conversely, tyrosol, 3,4-dihydroxyphenylacetic acid, and indole-3-acetic acid were downregulated in the 4% CPFF group.

### 3.6. Growth Performance

Table 10 summarizes the effects of CPFF on the growth performance of growing cattle. Although no significant differences were observed in initial or final body weight between the two groups (*p* > 0.05), the 4% CPFF group exhibited a significantly higher average daily gain (ADG) compared to the CON group (*p* = 0.004). CPFF supplementation also significantly increased average daily feed intake (ADFI) (*p* = 0.001). As evidenced by a reduction in the feed conversion ratio (FCR) from 25.25 in the CON group to 18.13 in the CPFF group (*p* = 0.003).

## 4. Discussion

This study demonstrates that the regulatory effects of CPFF on rumen fermentation in beef cattle are dose-dependent, with 4% supplementation identified as the optimal level. In the in vitro fermentation experiment, the 4% CPFF group achieved the highest 48-h gas production. These results, coupled with a significant increase in MCP, suggest that this dosage is most conducive to the rumen fermentation process. While appropriate doses of composite microecological preparations typically improve the rumen environment through the synergistic effects of lactic acid bacteria, lactate-utilizing bacteria, yeasts, and fungi, their efficacy does not necessarily increase linearly with the dosage. Instead, the outcome depends on the specific combination of microbial strains, substrate characteristics, and diet composition. This observation aligns with previous reviews on direct-fed microbials in ruminants [28]. Supporting this, studies on fermented total mixed rations have shown that fermentation significantly alters carbohydrate composition and gas production kinetics, often yielding non-linear responses [29]. Similarly, while active dry yeast supplementation can improve feed intake and rumen microbiota, not all dosages consistently enhance fermentation [30]. Consequently, the 4% CPFF group outperformed both the 2% and 8% groups in this study, suggesting that the benefits of CPFF rely on the supplementation dose. It should be noted that the microbial counts and product composition of CPFF were provided by the manufacturer and were not independently verified by our laboratory, which represents a limitation of the present study.

Regarding fermentation parameters, 4% CPFF significantly increased the concentrations of acetate, butyrate, and microbial crude protein (MCP), as well as the acetate-to-propionate ratio. However, the rumen pH remained stable. This indicates that the primary effect of CPFF is not to intensify acidifying fermentation but rather associated with microbial protein synthesis. Acetate is typically associated with the enhanced degradation of structural carbohydrates [31], while butyrate is essential for rumen epithelial energy supply and functional maintenance [32]. Furthermore, the rise in MCP suggests an improved efficiency in converting ammonia nitrogen into microbial protein, which directly enhances ruminal nitrogen utilization [33]. Similar shifts in fermentation and metabolic profiles were reported in Holstein steers supplemented with yeast fermentation products, where effects were attributed to coordinated microbial metabolism [34]. Changes in the concentrations and proportions of acetate and propionate in the rumen fluid indicated a shift in the fermentation pattern toward a fibrolytic profile. In this study, CPFF utilizes by-products like wheat bran and cottonseed meal as primary substrates. Moreover, the composite microbial inoculum includes fungi and Bacillus species, both of which theoretically favor cell wall degradation and acetate production. Therefore, the “increased acetate and relatively decreased propionate” pattern observed here aligns with the specific substrate background of this study. These results contrast with some studies reporting increased propionate and a decreased acetate-to-propionate ratio. Such discrepancies likely stem from differences in fermentation substrate composition and product properties [26]. Furthermore, no methane inhibition was observed in the 4% CPFF group. This could be attributed to the abundant fermentable substrates in CPFF, such as soluble carbohydrates and lactic acid derived from fermentation, which are rapidly degraded in the rumen to release hydrogen, thereby providing sufficient substrates for methanogens and promoting methanogenesis. This result is consistent with recent reviews regarding the relationship between rumen microbiota and methane emissions [35].

The in situ degradation results revealed that CPFF promoted fiber degradation while slowing down starch decomposition. Supplementation with 4% CPFF significantly increased the degradation rates and effective degradability (ED) of NDF and ADF at multiple time points. Conversely, the ED of starch significantly decreased. These findings indicate that CPFF does not simply accelerate the fermentation of all substrates. Instead, it optimizes the release rhythm of different nutrients, enhancing structural carbohydrate utilization while mitigating the metabolic fluctuations typically caused by rapid starch fermentation. Previous studies on beef heifers demonstrate that yeast culture supplementation can alter in situ degradation and fermentation responses, confirming that microecological regulation directly affects substrate kinetics [36,37]. Similarly, adding multi-fungal extracts to beef cattle diets improves fiber digestibility, which is recognized as a key mechanism for enhancing feed utilization [38]. The importance of optimizing local feed resources to enhance fiber utilization and overall productivity has been emphasized in recent studies [39]. Research on fermented palm kernel meal also indicates that substrates co-fermented with fungi and enzymes improve nutrient degradation and microbial adaptability [40]. The distinctive feature of the current study is the simultaneous observation of enhanced fiber degradation and a decreased starch degradation rate, alongside an increased acetate-to-propionate (A/P) ratio. This suggests that CPFF functions by optimizing the synchrony of energy release rather than merely accelerating overall fermentation. Research shows that for growing cattle fed a total mixed ration (TMR), this “sustained fiber fermentation + slow-release starch” profile is highly beneficial. It reduces rumen pH fluctuations and the risk of subacute rumen acidosis (SARA) caused by rapid starch fermentation, while improving the synergistic utilization of energy and nitrogen by rumen microorganisms [41,42]. Meanwhile, the addition of feeds with different starch degradation rates (e.g., bitter vetch and sorghum grain) affects the growth performance, carcass characteristics, fatty acid profile, and meat quality of male goats [43].

The microbiota sequencing results were highly consistent with the observed changes in degradation kinetics. In the 4% CPFF group, the dominance of specific taxa decreased, leading to a more even community distribution. This group was significantly enriched with *Rikenellaceae_RC9_gut_*group, *Christensenellaceae_R-7_*group, and *UCG-002*. *Christensenellaceae_R-7_*group and *UCG-002* are key bacteria responsible for fiber degradation in the rumen; therefore, their increase typically indicates an enhanced capacity to hydrolyze fibrous polysaccharides [44]. Similarly, *Rikenellaceae_RC9_gut_*group coexists with beneficial rumen bacteria to produce various short-chain fatty acids (SCFAs), which inhibits the proliferation of harmful microbes and helps maintain fermentation homeostasis [45]. These findings align with previous research. For instance, feeding fermented palm kernel meal to beef cattle increased Fibrobacteres levels, corresponding to an improved ability to utilize dietary protein, carbohydrates, and fiber [46]. Additionally, supplementing Brahman cattle diets with 20% yeast-fermented cassava roots has been shown to increase both total bacterial counts and neutral detergent fiber (NDF) digestibility [47].

Previous research has demonstrated that fermented feed mixtures, such as cotton stalk and apple pomace, can significantly alter the rumen microbiota and metabolome without disrupting microbial homeostasis [48]. Consistent with the present study, using microbial consortia in finishing cattle diets can influence feed intake, digestibility, and rumen dynamics. However, these microbial effects are heavily contingent upon the dietary forage-to-concentrate ratio [49,50]. A notable finding in this study is that increased microbiota diversity occurred concurrently with improved growth performance. While rumen microbiota patterns related to feed efficiency are highly influenced by diet composition, research suggests there is no universal “efficient microbiota template” applicable across all dietary conditions [51,52]. Consequently, CPFF likely enhanced the adaptability of the rumen ecosystem to complex substrates by increasing microbial diversity, which in turn strengthened fiber degradation capacity and supported overall performance.

The metabolomics results further support the microbiological findings at a functional level. CPFF supplementation caused a distinct separation in the rumen metabolic profile, with 95 differential metabolites identified. These metabolites were primarily enriched in the amino acid metabolism pathway, specifically tryptophan and tyrosine metabolism. The results of the correlation analysis indicate that formylanthranilic acid and indoxyl were upregulated and showed positive correlations with the genus *UCG-002*. Tryptophan metabolites, particularly indole compounds, are recognized as key molecules in host–microbe signaling, local immune regulation, and epithelial barrier homeostasis [53]. Similar plasma metabolome shifts were observed in steers fed high-concentrate diets supplemented with yeast fermentation products, suggesting that the influence of microecological preparations extends beyond rumen fermentation to affect the host’s overall metabolic status [34]. A review of host–rumen microbiota interactions highlights that the impact of the microbiota on production performance and environmental phenotypes is driven by the dual effects of structural community shifts and metabolic network reprogramming [54]. In the current study, the enrichment of fiber-degrading bacteria, the remodeling of amino acid metabolic pathways, and improved growth performance occurred simultaneously. This indicates that CPFF likely exerts its effects through a sequential process: improving substrate structure, enhancing fiber degradation, increasing beneficial metabolite production, and ultimately optimizing the internal rumen environment. Additionally, it should be noted that the intra-ruminal degradation kinetics in this study were evaluated using a sheep model. Although small ruminants serve as excellent and operationally feasible physiological models for preliminary ruminal assessments, directly extrapolating absolute degradation values to beef cattle carries inherent limitations. Consequently, caution should be exercised when applying our findings to beef cattle, and future validation studies specifically targeting the target species are warranted.

In terms of growth performance, 4% CPFF significantly increased average daily gain (ADG) and average daily feed intake (ADFI) while reducing the feed conversion ratio (FCR). Notably, the simultaneous changes in ADFI and FCR suggest that CPFF does not merely stimulate appetite; more critically, it enhances the efficiency of nutrient utilization. These findings are supported by various studies on microecological interventions. For instance, yeast culture supplementation has been shown to increase the total tract digestibility of dry matter, organic matter, and fiber, while tending to improve ADG [55]. Similarly, Bacillus-based probiotics can enhance fiber digestibility and production performance in cattle fed high-fiber diets [56]. While some interventions, such as multi-fungal extracts or fermented concentrates, improve fiber utilization or alter fermentation patterns without significantly impacting weight gain [38,57], others show broader benefits. For example, microbially fermented rice bran significantly improved rumen fermentation, increased Prevotella abundance, and boosted both nutrient efficiency and milk yield in dairy cows [58]. Collectively, these results demonstrate that using fermented feed to optimize cattle health and performance through beneficial bacteria and functional metabolites is a highly feasible feeding strategy.

In summary, under the conditions of this experiment, CPFF improves the growth performance of beef cattle through a multi-stage mechanism. First, an appropriate dose of the complex fermentation product enhances substrate structure and nutrient accessibility via exogenous pre-fermentation. Upon entering the rumen, it promotes the enrichment of functional microbial taxa—such as *Rikenellaceae_RC9_gut_*group, *Christensenellaceae_R-7_*group, and *UCG-002*—which enhances fiber degradation and maintains fermentation homeostasis. Furthermore, correlation analysis revealed that CPFF enhanced the interaction efficiency between microbes and metabolites by reshaping the tryptophan metabolism network, which may account for the improvements in FCR. However, this study has several limitations. However, several inherent limitations of this feeding trial should be acknowledged. First, a formal sample-size power analysis was not conducted prior to the study, which may limit the statistical power to detect subtle or small effect sizes between treatment groups. Second, although the 33-day feeding period was sufficient to evaluate short-term responses, it may not fully reflect the impacts on long-term growth performance. An extended feeding trial would be beneficial to evaluate the temporal sustainability of the observed responses. Additionally, while 16S sequencing and untargeted metabolomics reveal significant correlations, they cannot fully establish causal relationships between specific microbiota and metabolites. Future research utilizing multi-omics integration and stable isotope technology is necessary to validate these functional links.

## 5. Conclusions

In conclusion, CPFF demonstrates feeding advantages for regulating rumen fermentation in beef cattle, with 4% supplementation yielding the most effective results. Specifically, it promotes acetate- and butyrate-type fermentation, improves the ED of NDF and ADF, and moderates the rapid degradation of starch. Furthermore, CPFF significantly increases rumen microbiota diversity and remodels metabolic pathways. These physiological shifts ultimately translate into improved ADG and enhanced FCR. However, the study has limitations, and the mechanistic link between the in vitro/in situ results and in vivo growth performance needs to be further confirmed by directly measuring rumen fermentation parameters and digestibility.

## Figures and Tables

**Table 1:** Ingredient composition and nutrient levels of fermentation mixed raw materials and CPFF (DM Basis) %.

Items	Contents (%)	—
Corn bran	67.00	—
Rice bran	17.40	—
Cottonseed meal	15.00	—
Urea	0.30	—
Brown sugar	0.30	—
Total	100.00	—
Nutritional Levels ^(1)^	FRM	CPFF
DM	89.90	88.42
CP	17.73	20.31
ADF	18.03	12.63
NDF	30.87	18.11
EE	6.13	3.36
Ash	5.79	8.42
Ca	1.29	1.52

DM = dry matter; CP = crude protein; ADF = acid detergent fiber; NDF = neutral detergent fiber; EE = ether extract; FRM = fermentation mixed raw materials; ^(1)^ Nutrient levels were measured values.

**Table 2:** Ingredient composition and nutritional levels of concentrate feed (DM basis) %.

Ingredients	Content	Nutritional Level ^(2)^	Content (%)
Corn	58.00	Dry matter	88.01
Spray-Dried corn bran	11.77	Crude protein	20.77
Corn germ meal	10.00	ADF	6.78
Wheat bran	10.00	NDF	18.13
Cottonseed meal	3.33	Starch	38.77
Limestone	2.85	Crude fat	2.01
Ammoniated corn stover	2.00	Ash	4.78
Sodium chloride	0.80	Ca	0.87
Sodium bicarbonate	0.60	P	0.57
Premix ^(1)^	0.60		
Mold inhibitor	0.05		
Total	100.00		

DM = dry matter; ADF = acid detergent fiber; NDF = neutral detergent fiber; ^(1)^ The premix provided the following per kg of the diet: VA 1000 IU, VD 210 IU, VE 25 IU, Cu 12 mg, Fe 80 mg, Mn 25 mg, Zn 70 mg, I 1.0 mg, Se 0.36 mg. ^(2)^ Nutrient levels were measured values.

**Table 3:** Feed composition and nutrient composition (DM basis) %.

Items	Contents
Ingredient composition	
Alfalfa hay	30.01
Corn	39.90
Steam-flaked corn	7.78
Soybean meal	11.40
Safflower seed meal	5.22
Ammoniated corn stover	0.57
Fatty acid calcium	2.28
Stone powder	1.01
CaHPO_4_	0.31
Nacl	0.40
Premix ^(1)^	1.12
Total	100
Nutrient composition ^(2)^	
CP	19.81
NDF	25.99
ADF	18.16
Ca	0.98
P	0.73

^(1)^ The premix provided the following per kg of the diet: VA 1000 IU, VD 210 IU, VE 25 IU, Cu 12 mg, Fe 80 mg, Mn 25 mg, Zn 70 mg, I 1.0 mg, Se 0.36 mg. ^(2)^ All nutrient levels were measured values.

**Table 4:** Composition and nutritional levels of TMR diet for growing cattle (DM basis) %.

Ingredients	Content	Nutrient Levels ^(2)^	Content
Alfalfa	22.20	Dry matter	58.15
Wheat straw	22.20	Crude protein	14.06
Oat hay	11.16	Acid detergent fiber	32.31
Corn	24.44	Neutral detergent fiber	41.41
Wheat Bran	4.44	Starch	17.01
Soybean hulls	0.89	Crude fat	2.69
Soybean meal	6.67	Ash	10.16
Beet pulp pellets	0.89	Ca	1.38
Rice bran	4.44	P	0.32
Ammoniated corn straw	0.44		
Dicalcium phosphate	0.44		
Limestone	0.89		
Premix ^(1)^	0.89		
Total	100		

^(1)^ The premix provided the following per kg of the diet: VA 1000 IU, VD 210 IU, VE 25 IU, Cu 12 mg, Fe 80 mg, Mn 25 mg, Zn 70 mg, I 1.0 mg, Se 0.36 mg. ^(2)^ All nutrient levels were measured values.

**Table 5:** Effects of different doses of CPFF added to feed on rumen fermentation parameters.

Items	CON	2% CPFF	4% CPFF	8% CPFF	SEM	*p*-Value
DMD (% DM)	84.82	85.00	82.93	83.32	0.40	0.125
CH_4_ (%)	8.94 ^b^	11.22 ^a^	11.78 ^a^	8.78 ^b^	0.27	<0.001
pH	6.24	6.27	6.23	6.23	0.01	0.120
NH_3_-N (mg/dL)	11.25 ^bc^	12.54 ^b^	14.93 ^a^	9.79 c	0.45	<0.001
MCP (µg/mL)	2258.00 ^b^	3115.00 ^ab^	3458.00 ^a^	2624.00 ^ab^	150.90	0.016
GP (mL/g)	90.05 ^c^	108.40 ^ab^	113.10 ^a^	100.90 ^b^	1.99	<0.001
Lactic acid (mmol/L)	0.56	0.58	0.53	0.43	0.03	0.214
Acetate (mmol/L)	81.00	84.41	94.93	75.08	3.39	0.215
Propionate (mmol/L)	37.06	30.00	31.41	37.52	1.55	0.077
Isobutyric (mmol/L)	1.78	1.85	1.96	1.66	0.08	0.624
Butyric acid (mmol/L)	10.06 ^a^	11.18 ^a^	12.68 ^a^	8.27 ^b^	0.48	0.009
Isovalerate (mmol/L)	2.25 ^ab^	3.00 ^ab^	3.30 ^a^	2.23 ^b^	0.13	0.015
Valerate (mmol/L)	1.84	2.05	2.25	1.65	0.09	0.089
TVFA (mmol/L)	131.40	132.50	149.30	132.60	5.42	0.559
A/P	2.27 ^b^	2.82 ^ac^	2.85 ^a^	2.15 ^c^	0.06	<0.001
Acetate (%)	61.61 ^b^	63.75 ^a^	63.64 ^a^	60.85 ^c^	0.25	<0.001
Propionate (%)	27.18 ^a^	22.60 ^b^	22.34 ^b^	28.28 ^a^	0.50	<0.001
Isobutyrate (%)	1.23 ^b^	1.40 ^a^	1.39 ^a^	1.25 ^a^	0.02	0.010
Butyric acid (%)	6.96 ^b^	8.45 ^a^	8.64 ^a^	6.69 ^b^	0.17	<0.001
Isobutyric (%)	1.75 ^b^	2.26 ^a^	2.38 ^a^	1.68 ^b^	0.06	<0.001
Valerate (%)	1.27 ^b^	1.55 ^a^	1.61 ^a^	1.25 ^b^	0.03	<0.001

MCP = microbial protein; GP = gas production; A/P = acetate to propionate ratio; SEM = standard error of the mean. Within the same row, values sharing a common superscript or lacking one indicate no significant difference (*p* > 0.05). Conversely, different lowercase superscripts (a, b, c) denote significant differences among treatments within the same incubation time (*p* < 0.05). These conventions apply to all subsequent tables.

**Table 6:** Gas production parameters of in vitro fermentation (mL).

Items	CON	2% CPFF	4% CPFF	8% CPFF	SEM	*p*-Value
6 h	4.15 ^b^	18.25 ^a^	19.76 ^a^	5.75 ^b^	1.30	<0.001
9 h	23.45 ^b^	44.65 ^a^	44.95 ^a^	25.10 ^b^	2.50	<0.001
12 h	46.18 ^b^	65.28 ^a^	66.70 ^a^	49.54 ^b^	2.89	<0.001
24 h	85.70 ^b^	104.60 ^a^	107.60 ^a^	91.16 ^b^	4.40	<0.001
48 h	118.10 ^b^	136.42 ^a^	141.13 ^a^	122.48 ^b^	2.35	<0.001
B (mL/g)	126.83 ^b^	140.57 ^ab^	148.92 ^a^	123.65 ^b^	3.12	<0.001
c (%/h)	0.067 ^b^	0.080 ^ab^	0.088 ^a^	0.064 ^b^	0.004	0.002

B represents potential gas production; c represents fractional rate of gas production. Data are mean ± SEM (n = 8 per group). Two-way repeated-measures ANOVA revealed significant main effects of treatment (F (5,12) = 282.8, *p* < 0.0001) and time (F (1,12) = 64.38, *p* < 0.0001). No significant treatment × time interaction was observed (F (5,12) = 0.3558, *p* = 0.8688). Tukey’s post hoc test: values at the same time point with different superscript letters differ significantly (*p* < 0.05).

**Table 7:** Nutrient degradation rates of feed raw materials and the 4% CPFF (%).

Items	Times	CON	4% CPFF	SEM	*p*-Adjust
DM degradation rate	2	55.69	55.58	0.35	>0.9999
	4	57.89	55.50	0.75	0.613
	8	65.01	62.11	1.00	0.398
	12	69.63	75.79	1.57	0.004
	24	80.19	76.67	0.77	0.199
	48	84.72	89.00	1.14	0.072
	a	49.47	49.65	0.74	0.907
	b	38.08	42.70	1.89	0.249
	c	0.0758	0.0556	0.0071	0.184
	ED	70.94	71.46	0.24	0.311
Starch degradation rate	2	73.08	61.78	5.10	0.028
	4	76.44	67.32	3.79	0.062
	8	82.30	74.43	3.23	0.008
	12	89.06	89.81	1.91	0.701
	24	93.88	92.92	0.69	>0.999
	48	97.09	98.65	0.48	0.910
	a	49.75	45.00	3.09	0.485
	b	45.42	53.42	3.06	0.262
	c	0.2049	0.1194	0.0085	0.007
	ED	86.26	82.62	0.58	0.034
NDF degradation rate	2	14.71	18.30	1.14	0.132
	4	16.59	21.76	1.22	0.025
	8	17.96	23.98	1.40	0.014
	12	20.08	28.74	1.99	0.050
	24	26.45	28.99	0.74	0.132
	48	35.25	66.73	7.09	0.012
	a	10.75	16.14	0.53	0.007
	b	89.25	83.86	0.53	0.007
	c	0.0137	0.0152	0.0007	0.358
	ED	29.87	35.67	0.44	0.003
ADF degradation rate	2	17.40	13.50	1.24	0.028
	4	21.77	14.19	1.79	0.062
	8	22.33	16.96	1.53	0.008
	12	24.12	22.75	0.86	0.701
	24	29.57	28.99	2.46	>0.999
	48	36.13	59.47	5.26	0.910
	a	18.74	11.32	1.00	0.021
	b	51.63	88.68	8.38	0.092
	c	0.0150	0.0157	0.0022	0.884
	ED	29.38	32.52	0.40	0.017
CP degradation rate	2	71.88	63.22	2.43	0.028
	4	73.22	71.07	0.84	0.062
	8	77.51	76.26	0.59	0.008
	12	82.01	85.61	1.49	0.701
	24	88.52	89.61	0.84	>0.999
	48	91.88	95.08	0.84	0.910
	a	68.41	56.70	2.48	0.077
	b	25.01	38.05	1.96	0.029
	c	0.0630	0.1017	0.0114	0.165
	ED	82.35	82.03	0.31	0.643

a represents rapidly degradable fraction; b represents slowly degradable fraction; c represents rate of degradation of fraction b; ED = effective degradability; DM = dry matter; NDF = neutral detergent fiber; ADF = acid detergent fiber; CP = crude protein. Data are presented as mean ± SEM (n = 6 per group). Two-way repeated-measures ANOVA was performed for each nutrient indicator. Significant main effects of treatment (*p* < 0.001) were observed for all parameters. The main effect of time was significant for starch (*p* = 0.0009) but not for NDF, ADF, CP, or DM (*p* > 0.05). A significant treatment × time interaction (*p* < 0.05) was found for all indicators except NDF (*p* = 0.869).

**Table 8:** Alpha diversity indices of rumen microorganisms (n = 6 per group).

Items	CON	4% CPFF	SEM	*p*-Adjust
Shannon	5.83	6.62	0.26	0.015
Chao	2674.00	3188.56	63.54	0.016
Sobs	2663.50	3156.33	65.94	0.016
ACE	2705.18	3251.81	66.38	0.016
Coverage	0.99	0.99	0.0016	0.045
Simpson	0.05	0.01	0.0022	0.015

**Table 9:** Amino acid metabolic pathways and differential metabolites.

Metabolic Pathway	Metabolite	VIP	*p*-Adjust	Regulate
Tyrosine metabolism	Leucodopachrome	3.59	1.380	up
	Tyrosol	2.75	0.894	down
	3,4-dihydroxyphenylacetic Acid	1.53	0.954	down
	Rosmarinate	1.29	1.028	up
Tryptophan metabolism	Indoleacetic acid	1.73	1.058	up
	Formylanthranilic acid	1.72	1.052	up
	Indole-3-acetic Acid	1.67	0.935	down
	Indoxyl	1.59	1.046	up
	4-(2-Amino-3-hydroxyphenyl)-2,4-dioxobutanoic acid	2.04	1.063	up

**Table 10:** Effects of feeding CPFF on growth performance of growing cattle.

Items	CON	CPFF	SEM	*p*-Value
Initial weight (kg)	397.30	372.80	6.80	0.072
Final weight (kg)	420.60	407.40	6.63	0.333
ADG (kg/d)	0.49	0.72	0.04	0.004
ADFI (kg)	11.59	11.84	0.05	0.001
FCR ^(1)^	25.25	18.13	1.27	0.003

ADG = average daily gain; ADFI = average daily feed intake; ^(1)^ Feed Conversion Ratio (FCR): The ratio of dry matter intake (kg) to average daily gain (kg).

## Data Availability

The authors confirm that all data underlying the findings are fully available without restriction.
